# Isolation and identification of *Pithomyces sacchari* as a leaf spot pathogen of *Helianthus annuus* from Pakistan

**DOI:** 10.1038/s41598-022-25890-z

**Published:** 2022-12-20

**Authors:** Shazia Shafique, Alina Javed, Sobiya Shafique, Abrar Hussain, Rubab Rafique, Ayesha Mubarak

**Affiliations:** 1 Department of Plant Pathology, Faculty of Agricultural Sciences, University of the Punjab, Lahore, 54590 Pakistan; 2 Department of Botany, University of Education, Township Campus, Lahore, Pakistan

**Keywords:** Biological techniques, Biotechnology, Molecular biology

## Abstract

Sunflower (*Helianthus annuus* L.) is an important annual crop known for its edible oil. Sunflower is susceptible to many fungal diseases including rusts, rotting, mildews, and leaf spots that result in low crop yield. Presently, infected leaves with leaf spot disease symptoms were collected from Jallo Park, Botanical Garden; University of the Punjab, Canal road, and Johar Town, Lahore for pathogen/s isolation and identification. The identification was executed morphologically as well as genetically by nucleotide sequencing of rDNA using Internal spacer region (ITS) and Glyceraldehyde 3-phosphate dehydrogenase (GAPDH) primers. Morphological characters demonstrated a rapidly growing colony on MEA reaching 5.0–5.5 cm in diameter without zonation. The mycelial growth was rough and cottony white from the front and light pink from the reverse side. Conidia were brown, verruculose, and ellipsoidal with three to five transverse septations and one longitudinal septum ranging from 15 to 30 ± 2 µm in the broadest part. Conidiophores were long, branched, septate, 70–80 × 3–4 µm in size. Based on morphological characteristics, the pathogen was identified as *Pithomyces sacchari*. In genetic characterization BLAST analysis of the rDNA-ITS region of the pathogen exhibited maximum (100%) homology with other *P. sacchari* GenBank strains. Similarly, 99% homology was found with partial glyceraldehyde 3-phosphate dehydrogenase (GAPDH). To confirm the pathogenicity, Koch’s pathogenicity test was performed by inoculating artificial fungal suspension in pots and plate assays. The emergence of similar disease symptoms and re-isolation of the same pathogens verified Koch’s pathogenicity postulates. Conclusively this study confirms the identification of this novel pathogen of sunflowers and necessitates the quick development of management tools.

## Introduction

The sunflower was introduced in Pakistan as an oilseed crop about 40 years ago. The production and expansion of sunflowers on land are fluctuating due to the invention of socio-economic constraints^1^. The annual yield of sunflower oil is 1.3 tons/ha in Pakistan. It is famous for its beauty; and the valuable importance of food. Sunflower oil is healthy, and its seeds are nutritious for many foods^2^. Its seeds are rich in vitamins, minerals, magnesium, potassium, selenium, and iron. They help in the improvement of brainpower, digestion, and functioning of the cardiovascular system. Whereas sunflower seed oil provides more Vitamins E as compared to other oil-containing crops. It also contains fats (mono-saturated and unsaturated). It is used in the treatment of many diseases i.e., obesity, heart diseases, indigestion, and is used to lower the level of saturated fats^3^.

The scientists reported 90–100 different diseases of sunflower worldwide^4^. The major diseases caused by fungi include rusts, phoma black stem, verticillium wilt, anthracnose, downy mildew, and leaf spot. Mostly fungal pathogens are the main cause of deleterious diseases in sunflower and other plants^2, 4^. The most destructive disease of sunflower is the *Sclerotnia sclerotiorum* seedling, the stalk, and head rots^4^. Several soil-borne pathogenic fungi reported from the sunflower seeds are *Alternaria, Aspergillus, Cladosporium Fusarium, Drechslera,* and *Penicillium*^2, 4^. The production of crops can be increased by controlling the disease-causing pathogens with better management practices. The present work is focused on the leaf spot diseases of sunflower caused by fungal pathogens that are indirectly affecting the economy of Pakistan because for the control of leaf spot fungi of sunflower it is necessary to accurately identify the fungi associated with leaf spot disease.

## Results

The present study was designed to explore the leaf spot disease in *H. annuus* including the isolation and identification of pathogenic fungi responsible for leaf spots.

### Analysis of field survey

**Figure 1:**
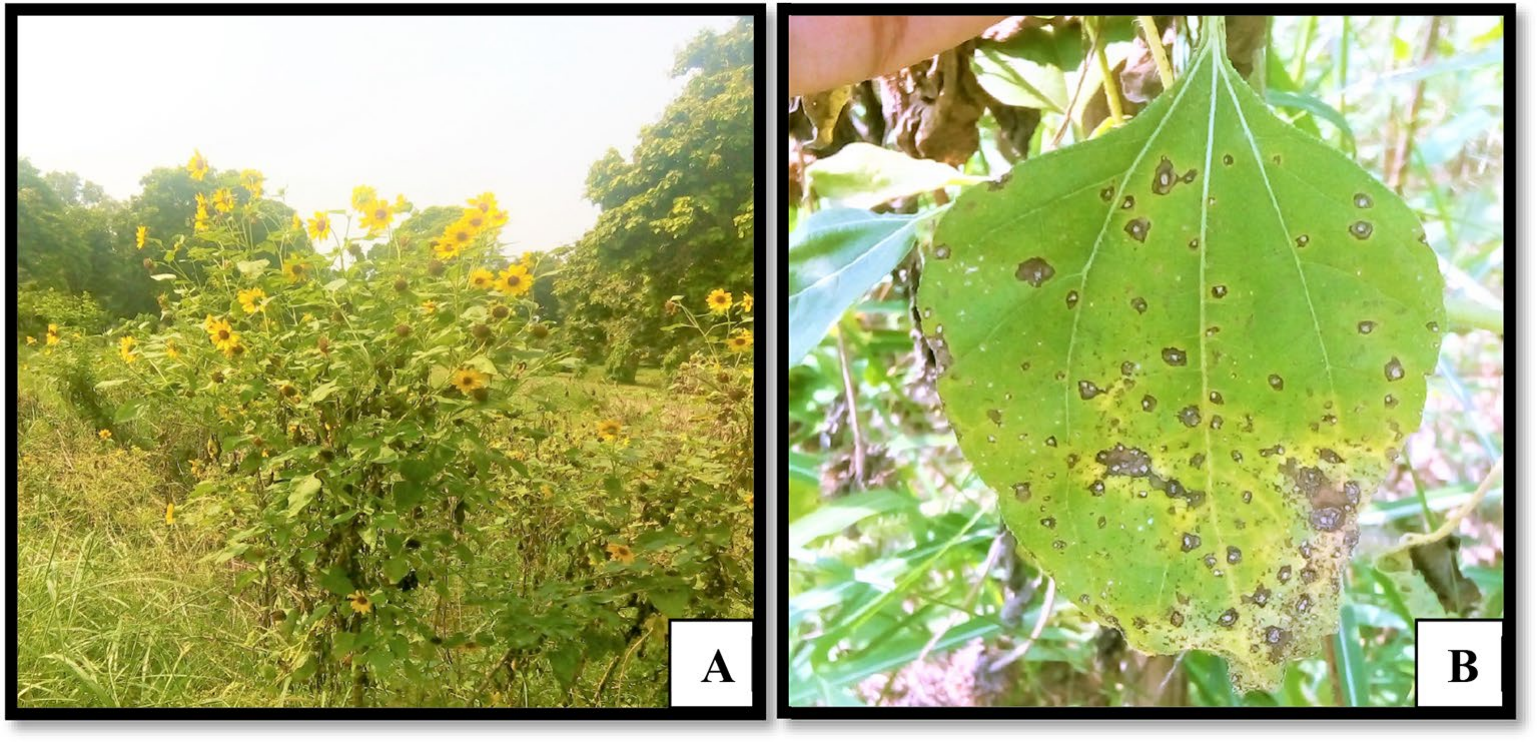
Survey of a diseased sunflower field. (**A**): Field view, (**B**): Infected sample.

**Figure 2:**
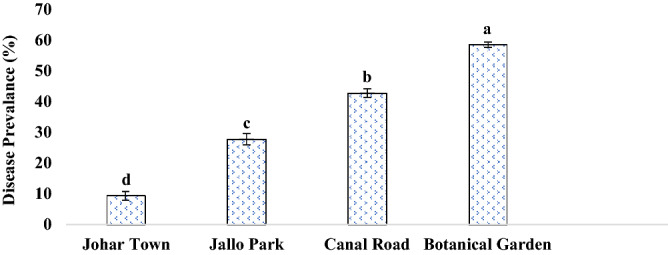
Disease Prevalence of different areas of Lahore. Vertical bars indicate standard errors of means of four replicates. Values with different letters show a significant difference by ANOVA (*p* ≤ 0.05) as determined by Statistix (ver. 8.1) software, LSD test at *p* = 0.05.

**Figure 3:**
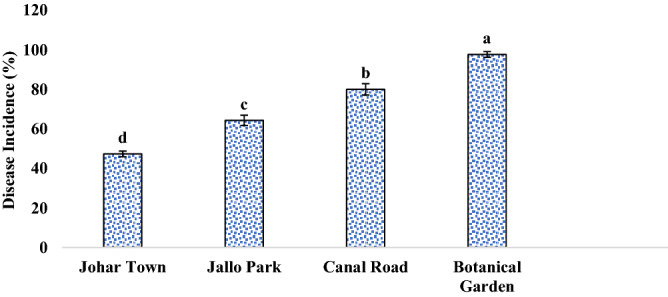
Disease incidence in different areas of Lahore. Vertical bars indicate standard errors of means of four replicates. Values with different letters show a significant difference by ANOVA (*p* ≤ 0.05) as determined by Statistix (ver. 8.1) software, LSD test at *p* = 0.05.

**Figure 4:**
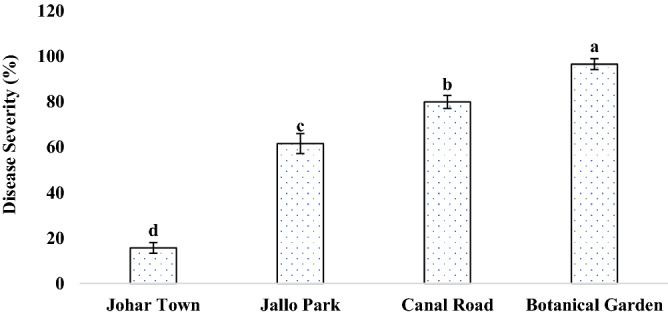
Disease severity of different areas of Lahore. Vertical bars indicate standard errors of means of four replicates. Values with different letters show a significant difference by ANOVA (*p* ≤ 0.05) as determined by Statistix (ver. 8.1) software, LSD test at *p* = 0.05.

A number of field surveys were conducted from June to July 2017–2018; in different areas of Lahore i.e., Jallo Park, Botanical Garden; PU, Canal road, and Johar Town, Lahore, and the infected samples were collected in every survey. The infected areas were observed, analyzed, and classified on the basis of disease prevalence, incidence, and severity (Figs. 1, 2, 3 and 4). During the survey, the samples collected from fields of sunflower were examined to be infected with leaf necrosis, lesions, and dead tissues. A field survey revealed more than 50% of *H. annuus* plants are infected with leaf necrosis. In general, the symptoms observed were brown irregular lesions with a yellow halo around them on leaves (Fig. 1). The size of the spots on leaves was 2–5 mm and about 40–50% of the leaf was found to be infected with such spots and lesions. Infected plants exhibited bad health while old infected leaves were found to be withered.

During the complete survey of Lahore district in consecutive years, disease prevalence was found in four to five places. Comparative analysis of these four locations revealed that the Botanical garden was the most infected area as the test plant was growing wildly there and it was attacked by a number of pathogens whereas Johar town was found to be least affected in terms of prevalence as well as the severity of the disease (Fig. 2). In terms of assessment of disease incidence during the survey, Botanical garden and Canal road were found to have the most infected and diseased plants i.e., among the existing plants there about; 97% and 80% of plants were infected; respectively (Fig. 3). When all these areas were evaluated for disease severity in plants, it was clearly noticed that maximum severity was recorded in the Botanical garden where more than 90% of leaves of a single plant were found to be infected from the abaxial and adaxial side with the signs of leaf spot disease (Fig. 4).

### Identification of the pathogen

**Figure 5:**
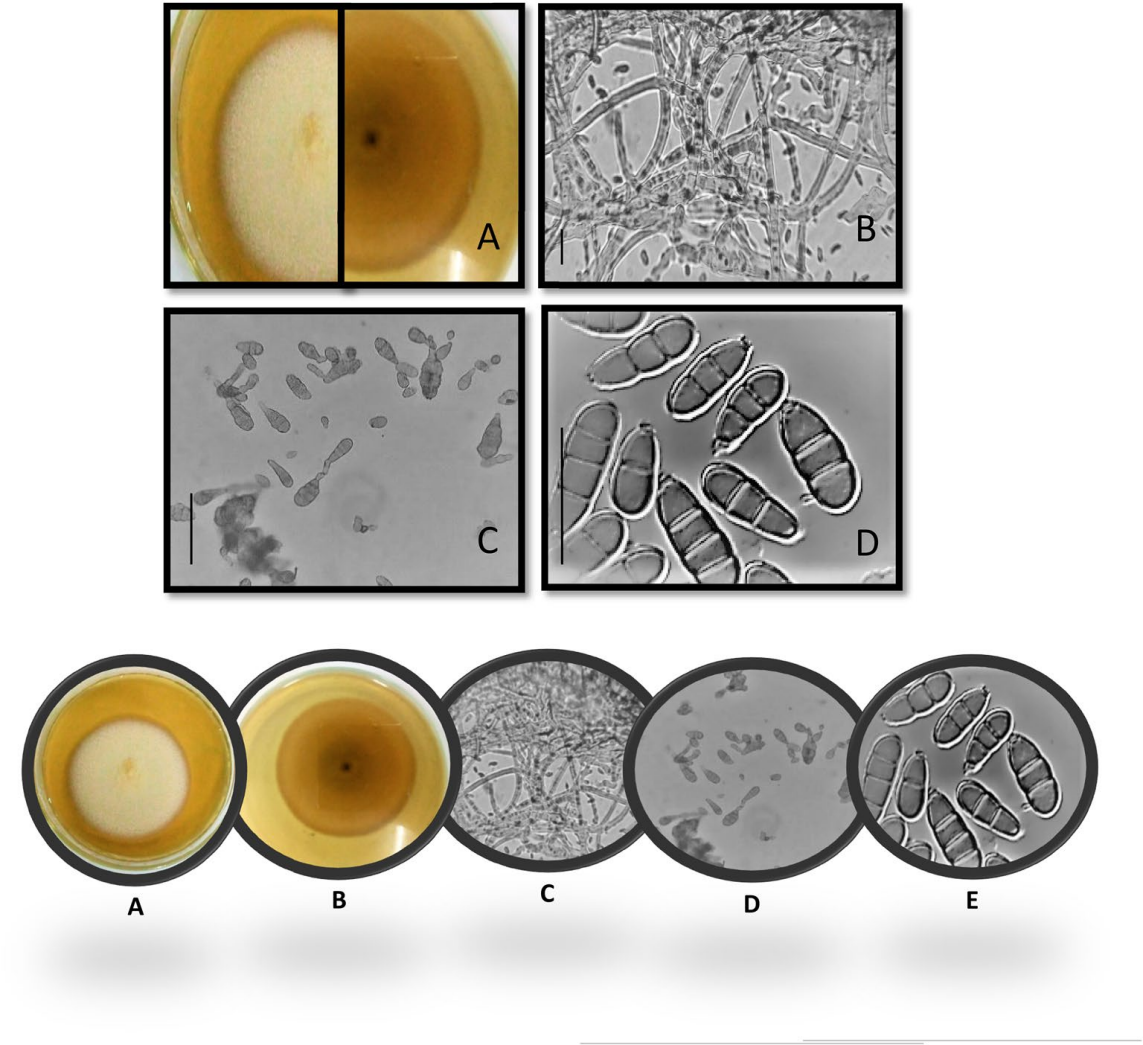
*Pithomyces sacchari* strain FB-103 isolated from leaf spots of sunflower. (**A**): Front and reverse of a colony grown on MEA; (B): Microphotograph of conidial heads at 10X; (**C**–**D**): Conidia at 40X and 100X magnification of a microscope, respectively. Scale bar: B = 5 µm, C = 10 µm and D = 20 µm.

Morphological identification studies of isolated pathogen revealed that the colony was growing rapidly on MEA reaching 5.0–5.5 cm in diameter without any zonation. The mycelial growth was rough and appeared cottony with white coloration from the front side and light pink from the reverse side (Fig. 5A & B). The microscopic features showed that the conidia were brown, verruculose, ellipsoidal, or slightly clavate with three to five transverse septations and one longitudinal septum ranging from 15 to 30 ± 2 µm in the broadest part. The spore wall was smooth but some had geniculations. The conidiophores were long, branched, septate, olive-brown, 70–80 × 3–4 µm in size (Fig. 5C–E). Based on morphological characteristics, the pathogen was identified as *Pithomyces sacchari* strain FB-103.

**Figure 6:**
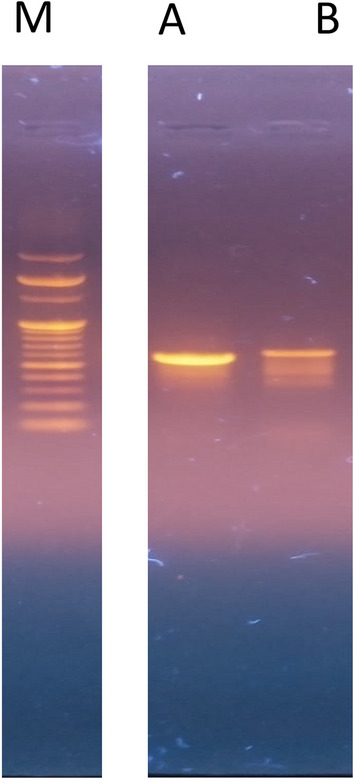
DNA fragments of *P. sacchari* strain FB-103 amplified by different primers; (**A**): Internal Transcribed Spacer sequences (ITS), (**B**): Glyceraldehyde 3–phosphate dehydrogenase (GAPDH), and (M): DNA Marker.

**Figure 7:**
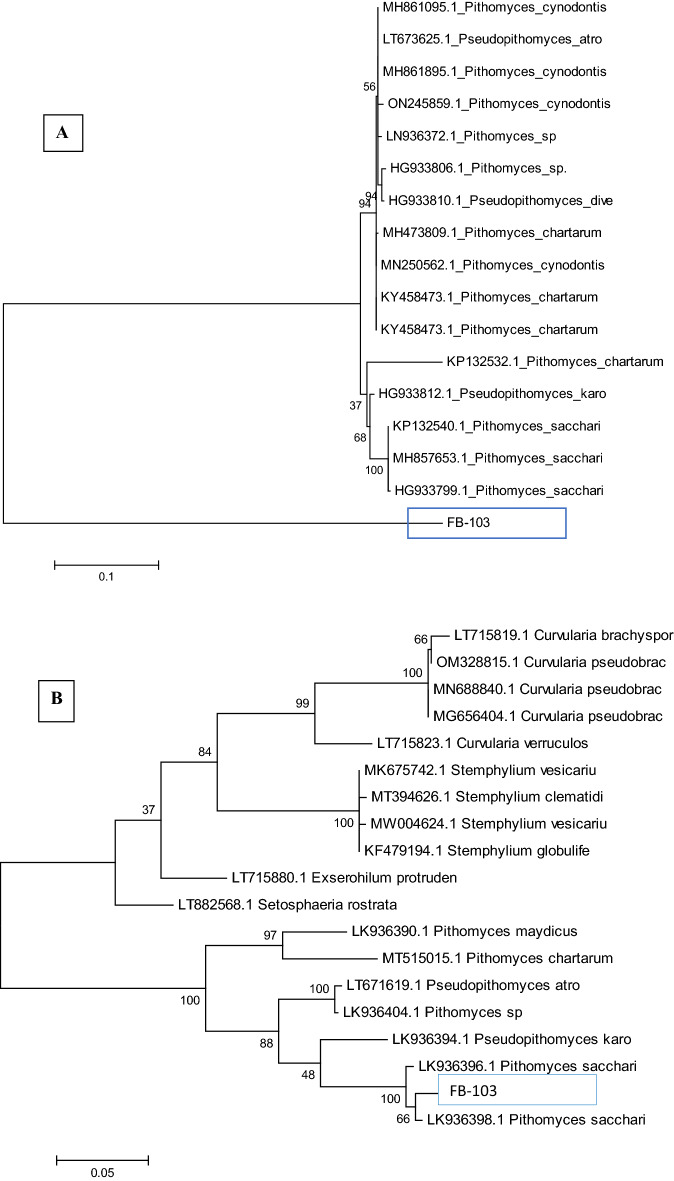
Phylogenetic tree of *P. sacchari* strain FB-103. (**A**): amplified by ITS Primer (**B**): amplified by GAPDH Primer.

Genetic characterization of identified species was done by sequence analysis of the ITS region by nucleotide BLAST analysis. The consensus primers ITS1 forward and ITS4 reverse successfully amplified the DNA fragment of total genomic DNA as a template. The band of 572 bp was shown on 1% agarose gel (Fig. 6A). The ITS sequence alignment of *P. sacchari* strain FB-103 showed 100% homology with MH857653.1, HG933799.1, and 99% similarity with KP132538.1 & KP132540.1. A phylogenetic tree representing the evolutionary relationship between the identified strain and other species was constructed (Fig. 7). The tree depicts branch lengths measured in the number of substitutions per site. The amplified ITS nucleotide sequence of *P. sacchari* strain FB-103 was assigned MN526025 accession ID in GenBank. Genetic characterization of morphologically identified species was performed by nucleotide sequence BLAST analysis of the rDNA–partial GAPDH gene. The primer successfully amplified the DNA fragment from total genomic DNA with a band of about 588 bp on 1% agarose gel (Fig. 6B). When the BLAST searches were carried out for the GAPDH sequence of *P. sacchari* strain FB-103, 99.03% similarity was found with *P. sacchari* LK936396.1, LK936398.1, and 98% similarity was detected with LK936397.1, LK936399.1. These sequences were used to build a phylogenetic tree, strain FB-103 shared the same branch with the strain of distant species of genus Pithomyces and a highly similar strain of *Pithomyces sacchari* as well. The percentage of trees in which the associated taxa clustered together is shown next to the branches. The amplified GADPH nucleotide sequence of *P. sacchari* strain FB-103 was assigned OP186486 accession ID in GenBank. All positions containing gaps and missing data were eliminated (Fig. 7).

### Analysis of pathogenicity test

**Figure 8:**
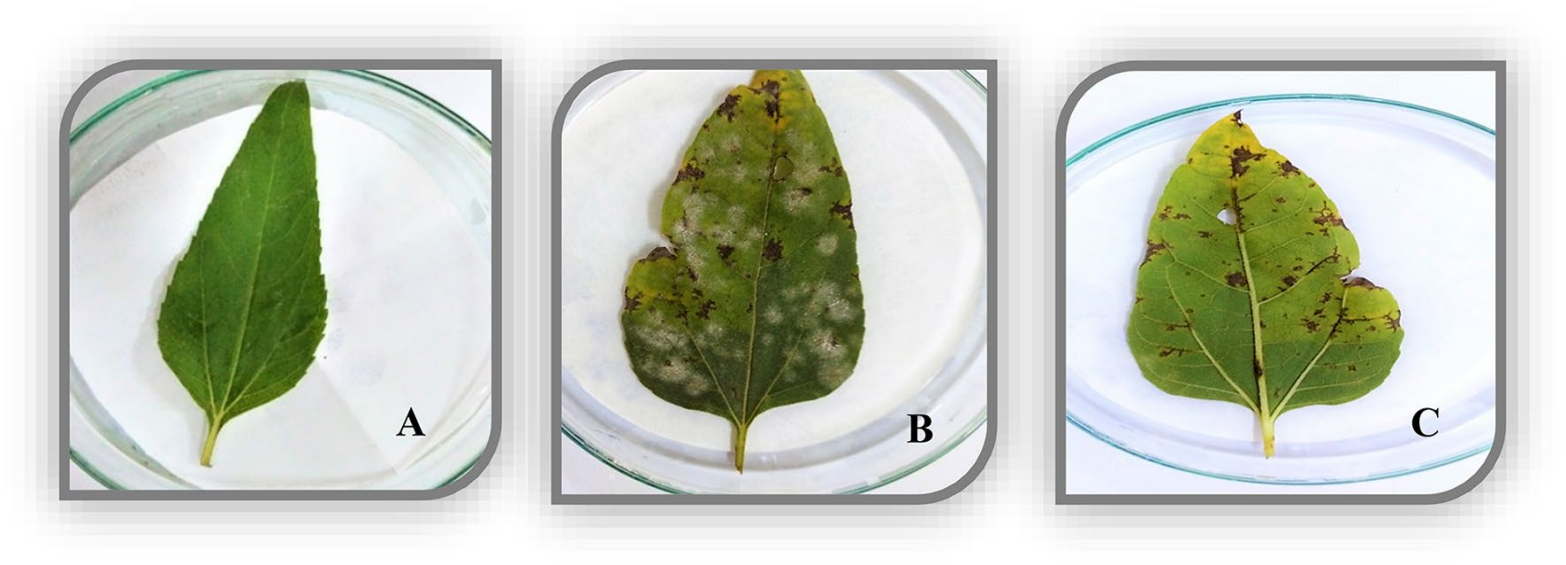
Comparative analysis of disease development caused by *Pithomyces sacchari* strain FB–03; (**A**): Control, (**B** & **C**): abaxial and adaxial side of the infected leaf, respectively.

The pathogenicity test was conducted to reconfirm the pathogen of a particular host and its pathogenic potential. This test was authorized by detached leaf assay in the laboratory as well as in vivo by giving artificial inoculation of pathogenic fungal spores to the host plants in the seedling stage. Results of the detached leaf method revealed that after 4–5 days of inoculation, the yellowing (chlorosis) started from the leaf tip and along the edges, while the midrib remained unaffected. Brownish and whitish spots appeared on the abaxial as well as the adaxial side of the leaf. Ultimately leaf spots caused necrosis, and holes in the leaves led to the rotting of leaves. Re-isolation of the pathogenic fungus from infected leaf tissues confirmed the disease etiology that ratified the same pathogen (Fig. 8).

**Figure 9:**
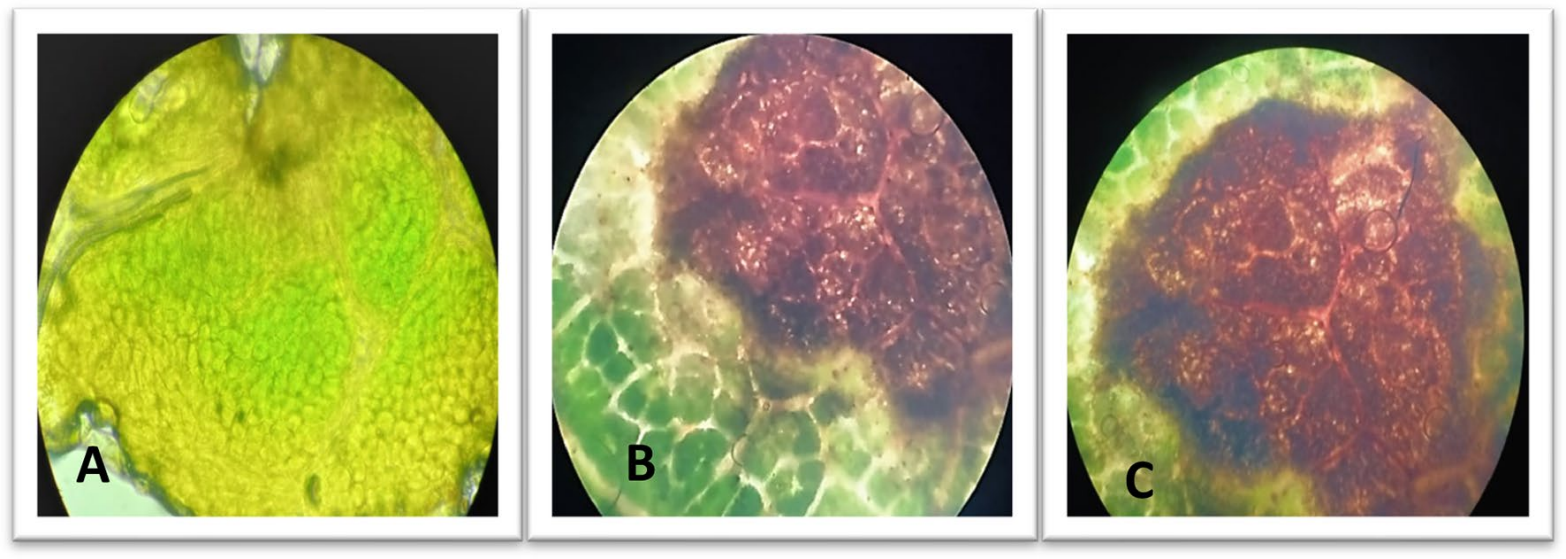
Micrographs of plant tissues showing histopathological studies on the development of infection by *Pithomyces sacchari* strain FB-103 in the host. (**A**): Control, host plant without infection (**B** & **C**): Development of infection in host plant at 10X and 40X, respectively.

This study was further extended to microscopic trials to detect the penetration of pathogens in the plant (Fig. 9). The interaction mechanism of *P. sacchari* strain FB-103 with sunflower was examined for up to 7 days by a combination of a light microscope (Labomed). The conidia caused swallowing and disintegration of chlorophyll. As the disease progressed, the number of conidial productions increased and more irregular patches of brown spots were observed. They have the ability to penetrate into mesophyll cells and grow parallel to the cuticle within epidermal cells. After severe infection cytoplasm of the cell and neighboring cell was disorganized, disrupted cell membrane, and damaged chloroplast was observed. Then ultimately infected cells of the host tissue started to collapse.

**Figure 10:**
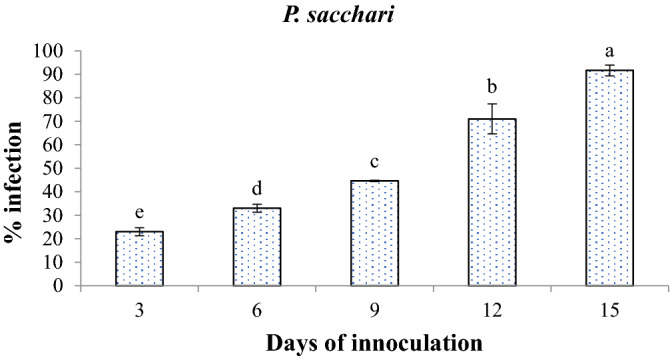
Comparative analysis of Disease severity of *P. sacchari* strain FB-103 on sunflower plant. Vertical bars show standard errors of the means of three replicates. Values with different letters show a significant difference by ANOVA (*p* ≤ 0.05) as determined by Statistix (ver. 8.1) software, HSD test at *p* = 0.05.

The in-vivo pathogenicity test was performed by growing seedlings in earthen pots. After 15 days, the seedlings that emerged were sprayed with 5 ml of spore suspension containing 5 × 10^5^ conidia/ml. Infection was evident in 8–10 days of post-inoculation. The symptoms observed on the plants were found to be similar to the samples that were collected during the survey. Foliar symptoms visualized were: yellowing followed by chlorotic spotting of the lowermost leaflets and in later stages, complete necrosis of leaves was noticed, and eventually, death of the plant occurred (Fig. 10). Data analysis of pot trials revealed that the pathogen was highly virulent and demonstrated 100% mortality in the host plant.

## Discussion

Sunflower is the most important crop for its oilseed and nutritional value. It is belonging to the family Asteraceae. It is affected by various fungal and bacterial diseases which result in a low yield of oil production^5–7^. The major fungal diseases are wilting, leaf spot, root rot, black stem, head rot, and downy mildew. Among all diseases leaf spot disease is a serious threat that affects the productivity of plants^8, 9^. Many leaf spot pathogens generally produce symptoms in leaf tissues wide-ranging in leaf spot ring size, lesions, color, and arrangement.

For the control of leaf spot fungi in sunflower, it is necessary to accurately identify the fungi associated with leaf spot disease. In the present study, sunflower leaf spots disease; caused by *Pithomyces sacchari* strain FB-103 was firstly reported from Pakistan. This study accordingly emphasized the precise identification of the agent causing leaf spots. Presently, the pathogen was isolated and identified on the basis of morphological and molecular characterization in which multilocus sequence analysis of internal transcribed spacers (ITS) and glyceraldehyde–3–phosphate dehydrogenase (GAPDH) coding genes using concerned primers and PCR products were obtained from total fungal genomic DNA. It has been widely accepted that ITS nucleotide sequences in combination with any coding gene, e.g. GAPDH, elongation factor, β-tubulin, OMD, calmodulin, etc. are a useful, easy and authentic way of fungal species identification^10^. In the contemporary lines, *Phoma herbarum* was isolated and identified in Pakistan from the necrotic areas of the leaves of *Cycas revoluta* on the basis of morphological characteristics followed by sequencing of the ITS region of its rDNA^11^. In another study, Shafique et al.^12^ conducted an experiment and identified the *Cladosporium cladosporioides* as the causal agent of the leaf spot of *Sonchus oleraceus.* They used rDNA spacer sequence and BLAST analysis which revealed *C. cladosporioides* as a leaf spot pathogen.

After pathogen identification; in a subsequent study, the pathogenic potential of *P. sacchari* strain FB-103 was assessed by applying Koch’s pathogenicity postulates using the leaf detached method and pot trials. The test pathogen proved very potent and induced characteristic symptoms like yellowing of leaves and dark brown spots on the leaves of sunflower plants along with whitish spots. In the parallel lines; Sahar ^13^ reported the pathogenic potential of *Setosphaeria rostrata, Cladosporium cladosporioides,* and *Curvularia clavata* on *Solanum melongena.* These three pathogens induced almost the same symptoms on the respective plant except for a few differences.

The present study concludes the report of novel isolation of leaf spot pathogen from sunflower plant and underlines the need for management of this pathogen which is responsible for yield loss of this important oilseed crop.

## Materials and methods

### Field survey and collection of samples

Sunflower is a wild and commonly grown plant species. A field survey was conducted to study and collect the samples of sunflower leaves infected with leaf spots from the fields of Botanical Garden, Canal road, JoharTown Lahore, and Jallo Park Lahore in 2017 and 2018. The plant was identified by Dr. Abdul Rehman Khan Niazi (Associate Professor, Institute of Botany, University of the Punjab, Lahore, Pakistan), assigned voucher no. LAH # 051,020 and was deposited in the Virtual Herbarium, Institute of Botany, University of the Punjab, Lahore, Pakistan. The latitude of the sampling place is 31.4° N and longitude is 74.35° E. The disease prevalence, incidence, and severity of affected area were calculated by the following forulae:

$${\text{Disease }}\;{\text{Prevalence }}\left( {{\% }} \right) = \frac{{{\text{Number }}\;{\text{of}}\;{\text{ diseased }}\;{\text{area }}\;{\text{observed}}}}{{{\text{Total }}\;{\text{number }}\;{\text{of }}\;{\text{area}}}}{ } \times 100$$

$${\text{Disease}}\;{\text{ Incidence }}\left( {{\% }} \right) = \frac{{{\text{Number}}\;{\text{ of}}\;{\text{ diseased}}\;{\text{ plants}}}}{{{\text{Total }}\;{\text{number }}\;{\text{of }}\;{\text{sample}}\;{\text{ plants}}}}{ } \times 100$$

$${\text{Disease}}\;{\text{ Severity }}\left( {{\% }} \right) = \frac{{{\text{Area }}\;{\text{of }}\;{\text{plant}}\;{\text{ part}}\;{\text{ affected}}}}{{{\text{Total }}\;{\text{leaf}}\;{\text{ area}}}}{ } \times 100$$

All the experiments on plants were carried out in accordance with guidelines obtained from publications of the Faculty of Agricultural Sciences, University of the Punjab, Lahore. For the isolation of the pathogen, in each survey, randomly five infected leaf samples per plant were chosen from each area, taken in sterilized polythene bags, brought to the laboratory, and saved at 4 °C for further experiments.

### Identification of the pathogen

#### Morphological identification

Fungal growth medium Malt Extract Agar (MEA) (2% malt extract and 2% agar, pH 6.5) was prepared for the isolation and purification of the pathogen^14^. For this, 4–5 spotted pieces of about 2 mm^2^ were cut from infected leaf samples which also contain some healthy parts of the leaf. These leaves were surface sterilized with 1% sodium hypochlorite solution for 5 min, followed by washing with distilled water. These pieces were inoculated on an MEA medium under aseptic conditions. The plates were incubated at 25 ± 2 °C. The Mycelium of the fungus coming out of inoculated leaf pieces was transferred to the new MEA plates and allowed to grow for the purification of fungal cultures. Pure cultures were placed at 4 °C for further experiments. The pure 7 days old fungus was initially identified on the basis of morphogenic characteristics. The colony characters observed under the stereoscope were; color, nature of culture from the forward and revere side, number of growth zones, the diameter of the colony, type of conidiophores, and chains of conidia. The microscopic characters recorded were; number, the position of septa (longitudinal and transverse) of conidia, type of attachment with the branch, spore size, number, spore wall, presence of beak, and shape. Pure culture of the isolated fungal pathogen was deposited at the First Fungal Culture Bank of Pakistan (FCBP), Institute of Agricultural Sciences, University of the Punjab, Lahore. The culture was assigned the number strain FB-103.

#### Molecular identification

Then purified culture was further identified or confirmed from nucleotide sequencing by using two primers of Internal Transcribed Spacer (ITS) sequence of rDNA and partial glyceraldehyde-3-phosphate dehydrogenase (GAPDH) genes. The detail of primers used for the identification of fungal pathogens in the present study is given in Table 1. The amplified PCR products were sent for sequencing and the results were analyzed by nucleotide Basic Local Alignment Search Tool (BLAST) analysis. Homology of sequences was checked with corresponding strains in GenBank database and used for the identification of fungi. Bio-edit (Ver. 7.2) software was used for NCBI blast analysis. All the sequence alignment files were downloaded with FASTA format and submitted to the Multiple sequence alignment tool using MUSCLE. Then the maximum likelihood trees were constructed using MEGA (ver. 6) software. Phylogenetic distances were estimated by the method of Jukes and Cantor. The tree topology was inferred by the neighbour-joining method with a 500-bootstrap value. The analysis involved 29 nucleotide sequences with ITS primer and 17 nucleotide sequences with GAPDH.

**Table 1:** Detail of primers used for identification of fungal pathogens in the present study.

Sr. no.	Gene	Primer name	Sequence (5'–3')	References
1	Internal Transcribed Spacer Region	ITS1 (Forward) ITS4 (Reverse)	5ʹ-TCC GTA GGT GAA CCT GCG G-3ʹ 5ʹ-TCC TCC GCT TAT TGA TAT GC-3ʹ	^13^
2	Glyceraldehyde-3-phosphate dehydrogenase	GAPDH (Forward) GAPDH (Reverse)	5ʹ-CAA CGG CTT CGG TGG CAT TG-3ʹ 5ʹ-GCC AAG CAG TTG GTT GTG C-3ʹ	^15^

### Pathogenic potential of isolated pathogen

In further studies, the pathogenic potential of the isolated virulent fungus was validated in three consecutive years (2017–2019) by detached leaf assay in the laboratory as well as in vivo by giving artificial inoculation of pathogenic fungal spores to the host plants in the seedling stage. The spore suspension (5 × 10^5^ spores/ml) was prepared to be used as inoculum under aseptic conditions. For the detached leaf method, sterilized Petri plates were taken and two-layer filter papers were placed in each Petri plate and moistened with doubled distilled water. The leaves were placed on a plate in such a way that their petiole touched the surface of moistened filter paper. The inoculum (2 ml) was provided on leaves and examined regularly for the emergence of symptoms. Control received the same amount of distilled water. There were eight replicate plates in each trail. The plates were incubated at room temperature (25–28 °C) with 42% humidity. After 7–8 days, the infected part was observed, compared with the sample collected from fields, and re-isolated to confirm Koch’s postulates.

For pot trials, earthen pots were taken, washed with water properly, and filled with soil at the rate of 2 kg per pot. Then sunflower seeds were sown and the pots were placed in the growth room at 24–28 °C. In each pot trail, eight replicates were employed with three plants in each pot, and the humidity was maintained at 42%. For the confirmation of Koch’s postulates, a pathogenicity test was set up by injecting a 5 ml spore suspension containing 5 × 10^5^ spores/ml with the help of a sterilized syringe in the stem nodes and also by the spraying of spore suspension in soil. Control received the same amount of distilled water. The plants were covered with polythene bags for 24 h for the maintenance of the spore germination and disease emergence. After 2 days of inoculation, the plants were transferred in shade under optimum temperature i.e. 25–28 °C and watered properly. These were examined regularly to evaluate the symptoms development. The pathogen was re-identified as *Pithomyces sacchari* strain FB-103.

## Data Availability

The data that support the findings of this study are contained within the manuscript. All the sequences used in this study have been successfully uploaded to the NCBI repository. The nucleotide sequences obtained with ITS and GAPDH have been uploaded successfully under the accession IDs MN526025 and OP186486; respectively.
